# Susceptibility and Mechanism of Aflatoxin Contamination of *Ziziphus jujuba* var. *spinosa*

**DOI:** 10.3390/toxins17030113

**Published:** 2025-02-27

**Authors:** Abdelrahman Elamin, Shohei Sakuda

**Affiliations:** Department of Biosciences, Teikyo University, 1-1 Toyosatodai, Utsunomiya 320-8551, Tochigi, Japan; a.elamin@nasu.bio.teikyo-u.ac.jp

**Keywords:** jujube seed, food supplement, aflatoxins, hilar region, HPLC, SEM

## Abstract

The susceptibility and mechanism of aflatoxin (AF) contamination in *Ziziphus jujuba* var. *spinosa*, whose seeds are important for medicinal use, were evaluated in this study. First, the susceptibility of intact fruits, classified into four maturity groups, to AF accumulation was assessed through artificial contamination with an aflatoxigenic *Aspergillus flavus* strain. AF analysis revealed that mid-mature fruits were highly susceptible to AF contamination. Next, AF accumulation in seed parts was examined by artificially inoculating *A. flavus* on intact fruits, showing AF presence in seeds after 30 days of incubation. The susceptibility of jujube kernels to AF accumulation in seed parts was then studied. The artificial inoculation of *A. flavus* on kernels, classified into three groups based on the pedicel condition, showed no correlation between AF contamination and the pedicel condition, with large fluctuations within each group. Finally, the effect of the hilar region morphology on AF contamination in seeds was investigated. The microscopic investigation of artificially contaminated seeds and AF quantification revealed that variations in AF concentration were linked to differences in the hilar region morphology.

## 1. Introduction

Jujube fruit is widely used as food and a dietary supplement due to its rich phytochemical content, including triterpene acids and their saponins, unsaturated fatty acids, flavonoid *C*-glycosides, alkaloids, and indole derivatives, which promote a healthy diet [1,2,3]. Among jujube varieties, *Ziziphus jujuba* var. *spinosa* is notable for its medicinal seed, known as “Sansonin” in Japanese, which exhibits anti-anxiety and antidepressant properties [4].

Mycotoxins are toxic secondary metabolites produced by various fungal species, including *Aspergillus*, *Penicillium*, *Fusarium*, *Claviceps*, and *Alternaria*. With around 400 known compounds, mycotoxins are widespread in agricultural commodities and medicinal herbs across diverse regions [5]. Among those posing the greatest threat to medicinal herbs are aflatoxins (AFs), ochratoxins (OTs), fumonisins, zearalenone, and deoxynivalenol [6].

AFs are the most commonly detected mycotoxins in crude medicinal plants. Primarily produced by *Aspergillus flavus* and *Aspergillus parasiticus*, AFs include four major types: aflatoxin B_1_ (AFB_1_), aflatoxin B_2_ (AFB_2_), aflatoxin G_1_ (AFG_1_), and aflatoxin G_2_ (AFG_2_) [7]. Recognized as Group 1 carcinogens by the International Agency for Research on Cancer (IARC), AFs are highly carcinogenic to humans [8].

Several studies have reported fungal infection and AF contamination in jujube fruits [9,10,11,12]. Aflatoxigenic *A. flavus* strains have been isolated from fruits in Iraq, preharvest fruits in India, and AF-contaminated fruits in Zambian markets. Ripe fruits in Bangkok showed AF contamination at 2.5–6.1 ppb. Due to the lack of basic studies on AF contamination prevention in jujube fruits, we conducted artificial inoculation experiments to assess the susceptibility of *Z. jujuba* var. *spinosa* to AF contamination, which we preliminarily reported in 2021 [4]. Our experiments revealed that mid-mature fruits were highly susceptible to AF contamination when *A. flavus* was inoculated on artificially wounded fruits. After cultivation, pooled fruits at the same maturity stage were analyzed for AF content. To confirm the susceptibility findings, additional experiments using nonwounded fruits and AF analysis in individual fruits are necessary.

Because jujube seeds are used in medicine, understanding AF contamination mechanisms is crucial for prevention. Our previous report speculated, based on microscopic observations, that fungal mycelial penetration occurs through the seed hilar region. However, the relationship between the hilar region morphology and AF contamination levels remains unclear.

Therefore, this study aims to evaluate (1) the susceptibility of intact jujube fruits at different maturity stages to AF contamination and the possibility of AF accumulation in seed parts; (2) the effect of pericarp layers (seed-surrounding layers) on AF accumulation in jujube seeds; and (3) the role of hilar region morphology in fungal mycelial penetration and AF accumulation in jujube seeds.

## 2. Results

### 2.1. Susceptibility of Intact Jujube Fruits at Different Stages of Maturity to AF Contamination

The HPLC analysis (Table 1) of the AFB_1_ and AFB_2_ levels in whole fruits revealed high AF accumulation in GBG (mid-mature) fruits after 15 and 30 days of incubation and in BG (mid-mature) fruits after 30 days. In contrast, GG (immature) and DBG (mature) fruits showed little or no AF contamination. The high susceptibility of mid-mature fruits to AF contamination was consistent with our previous experiments using artificially wounded fruits and pooled samples. However, individual fruit analysis revealed large variations in AF concentrations within the same maturity group. For instance, the AFB_1_ concentrations in GBG fruits after 15 days of incubation ranged from 0.7 to 3499 µg/kg.

### 2.2. AF Accumulation in the Seed Parts of Mid-Mature Fruits

To confirm the AF accumulation in seed parts, *A. flavus* was artificially inoculated on intact GBG fruits using the same method as in Section 2.1. After 30 days of incubation, the AF levels in the combined exocarp and mesocarp, as well as in seed parts, were analyzed (Table 2). Among 12 fruits, AF accumulation was detected in the combined parts of 7 fruits and the seeds of 3 fruits, indicating that AF can accumulate in seeds after 30 days. In two replicates (No. 2 and 11), the AF concentrations were higher in seeds than in the combined parts. Large fluctuations in the AF levels among fruits were reproduced.

### 2.3. Susceptibility of Jujube Kernels to AF Accumulation in Their Seed Parts

Jujube seeds are enclosed by three layers: the exocarp, mesocarp, and endocarp [13]. The endocarp plays a crucial role in protecting the seed [14]. To examine its effect on the susceptibility of seeds to AF contamination, GBG fruits were divided into three subgroups: GBG-A (fruits with pedicels), GBG-B (fruits without pedicels), and GBG-C (fruits without pedicels that were artificially damaged at the pedicel contact area). The exocarp and mesocarp were removed to prepare kernels. Five kernels per group were inoculated with *A. flavus* spores at the pedicel area and incubated for 5 or 10 days. The AF concentrations in the endocarp and seed of each kernel were analyzed (Table 3). AF contamination in the seed was observed in two GBG-B and one GBG-C kernels after 5 days and in two GBG-A kernels and one GBG-C kernel after 10 days. This indicates that AF can accumulate in seeds within 5–10 days, but that the pedicel area conditions did not affect the AF levels. After 10 days, only two seeds, Rep. 4 of GBG-A and Rep. 2 of GBG-C, exhibited exceedingly high AF concentrations, highlighting large fluctuations in the AF levels among seeds.

When GBG-B kernels were inoculated by dipping them in *A. flavus* spore suspension and incubated for 30 days, AF accumulation in seeds was observed in 4 out of 10 kernels (Table 4), further confirming large variations in the AF concentrations among seeds.

### 2.4. Relationship of the Hilar Region Morphology with the AF Contamination Level in the Seed

Since the seed hilar region is the primary entry point for water and can become permeable under environmental stresses such as microbial attack [4], fluctuations in the AF concentrations in seeds may be linked to the response of the hilar region to fungal mycelial stress. To investigate this, a microscopic study was conducted to assess differences in the hilar region morphology and their effect on fungal penetration and AF accumulation.

**Figure 1:**
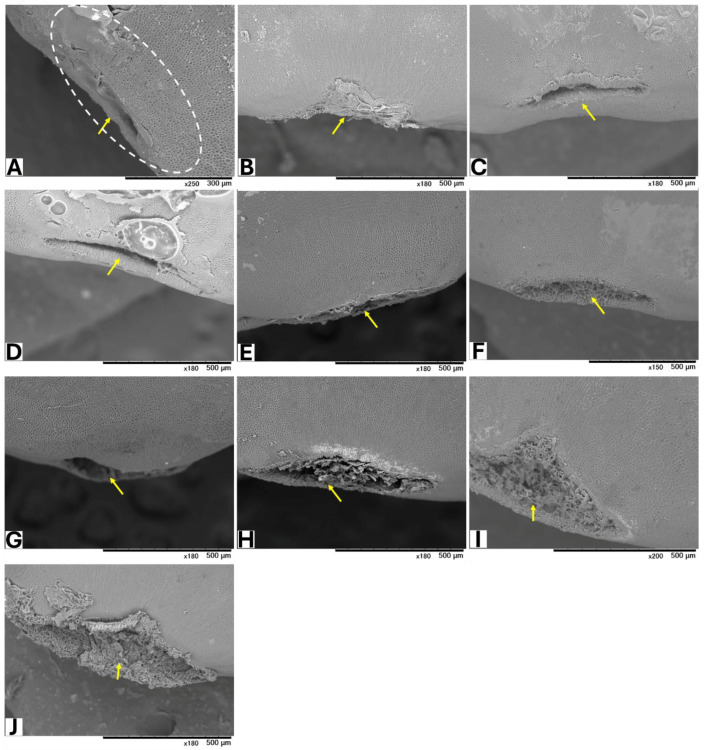
SEM images of seeds without inoculation of *A. flavus*. The white dotted circle denotes the HR (hilar region). Yellow arrows show HF (hilar fissure).

**Figure 2:**
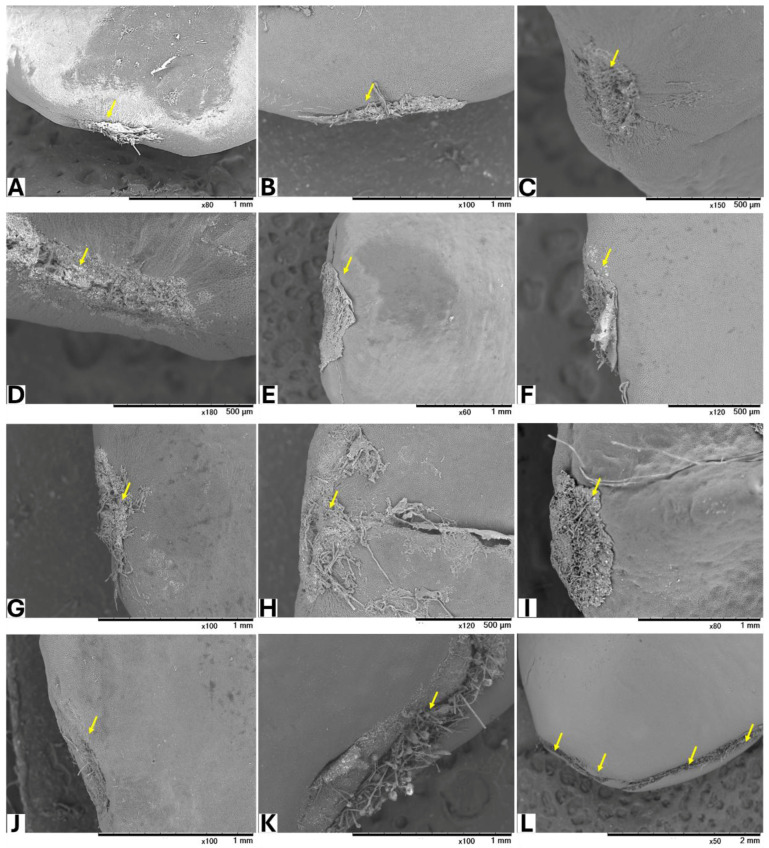
SEM images of seeds incubated with *A. flavus*. Yellow arrows show HF of the seeds after removing the accumulated fungal mycelia.

Seeds from GBG fruits were placed on modified CZA, with or without *A. flavus* spores, and incubated at 25 °C for 5 days. Based on a visual assessment of SEM images, three classifications of hilar fissure morphology in seeds incubated without spores were revealed (Figure 1): nearly closed (Figure 1A–C), semi-closed (Figure 1D–F), and open (Figure 1G–J). A similar classification was observed in seeds incubated with spores (Figure 2): nearly closed (Figure 2A–F), semi-closed (Figure 2G–K), and open (Figure 2L). The AF concentrations in the seeds (Table 5) appeared to correlate with the hilar fissure classification. The accumulation of fungal mycelia, which formed infection pads on the hilar fissure (Figure 2), increased with the width of the fissure opening.

## 3. Discussion

Jujube fruit is one of the most widely used medicinal herbs globally, and AF contamination [11,12] has raised significant concerns in the herbal medicine industry. Studies have explored the relationship between fruit maturation, nutrient concentrations, and AF levels, showing that unsaturated fatty acids, soluble sugars, and asparagine may affect susceptibility to AF contamination [4,15,16,17,18].

Our previous study [4] evaluated AF susceptibility in whole, wounded jujube fruits and their separate parts at different maturity stages, concluding that mid-mature wounded fruits and their seed parts are the most susceptible. This study aimed to determine whether AFs can also contaminate intact fruits and kernels and accumulate in seeds. Intact fruits protect their seeds through a pericarp composed of three layers: the exocarp, mesocarp, and endocarp [13]. The exocarp consists mainly of cuticle and epidermal cells, whose shape, size, and arrangement vary according to the stage of maturation, changing from compact to loose as the fruit matures [19]. The epidermal cell thickness decreases with maturation, while the pericarp cellulose content declines as lignin and hemicellulose levels increase [18]. The endocarp hardens through secondary cell wall formation and lignification, crucial for resistance to biotic stress [19]. Understanding fungal penetration through these layers to accumulate aflatoxins (AF) in jujube seeds is essential [20].

In this study, intact fruits at different maturity stages (Figure S1) were inoculated with *A. flavus* spores and incubated for 15 and 30 days. Consistent with previous findings [4], mid-mature fruits were the most susceptible to AF contamination, while mature fruits were least susceptible. However, mid-mature (GBG) replicates exhibited fluctuating AF concentrations, suggesting a variation in *A. flavus* mycelial penetration [21,22].

To confirm the fungal penetration and AF accumulation in seeds of intact fruits, mid-mature fruits were inoculated with *A. flavus* and incubated for 30 days. Each replicate was divided into seed and combined exocarp-mesocarp parts. AF analysis confirmed AF accumulation in seeds and reproduced fluctuations in AF levels.

Since the endocarp protects the seed and the pedicel is the only connection between the seed and the external environment, mid-mature (GBG) fruits were divided into three subgroups to examine mycelial penetration (Figure S2). The exocarp and mesocarp were removed, leaving only the kernels (endocarp with seeds). The pedicels were left intact in GBG-A, removed in GBG-B, and artificially damaged in GBG-C. After incubation, AF analysis unexpectedly showed the highest AF accumulation in a GBG-A replicate, suggesting that subgroup preparation did not influence the AF levels. Fluctuations in the AF concentrations were observed, consistent with previous fruit experiments. A separate experiment, in which mid-mature kernels were dipped in an *A. flavus* spore suspension and incubated for 30 days, reproduced these fluctuations.

Microscopic observations of control and artificially contaminated GBG seeds revealed differences in the hilar region (HR) and hilar fissure (HF) morphology. AF concentrations correlated with the HF width, which naturally varies among seeds. This aligns with studies on jujube seed structure and physical characteristics. *Z. jujuba* seeds are water-impermeable and exhibit physical dormancy [23]. The seed coat (SC) has a thick, hydrophobic palisade cell layer, making it impermeable. However, the HR differs structurally from the SC [24,25] and can become permeable under fungal attack [4]. Fungal penetration in intact plants [26] involves hyphal accumulation forming infection pads that modify the morphology and generate pressure to aid penetration. Variations in the seed structure and infection pad stress of jujube may explain the differences in the HF response and, consequently, the variability in AF accumulation.

## 4. Conclusions

Whole mid-mature jujube fruits and their seeds are highly susceptible to AF contamination, making this stage more vulnerable than others. Despite protective barriers (exocarp, mesocarp, and endocarp), aflatoxigenic fungi can penetrate intact fruit and kernels. Once inside, the fungi colonize the seed, leading to significant AF accumulation. The AF levels in seeds are influenced by variations in the hilar region’s morphology, which differ among seeds of the same maturity. This highlights the complex relationship between seed structure and fungal pathogenicity, suggesting that morphological variations in the hilar region significantly impact susceptibility to fungal invasion and AF accumulation.

## 5. Materials and Methods

### 5.1. Samples Preparation

The fruits of *Z. jujuba* var. *spinosa* were collected from multiple trees in a field in Ibaraki, Japan, in October 2022–2024. Intact fruits, kernels and seeds were evaluated in five experiments to determine the susceptibility of the seeds of jujube fruits to AF contamination and its mechanism.

#### 5.1.1. Intact Jujube Fruits and Jujube Kernels

Intact jujube fruits at different maturity stages were selected and classified into four color-based groups: green (GG), greenish-brown (GBG), brown (BG), and dark brown (DBG). Because the selected fruits inconsistently included pedicels, any attached pedicels were removed. Each fruit group was washed with ethanol:water (1:1 *v*/*v*), placed in autoclaved beakers (Figure S1), and stored in a biosafety cabinet overnight to allow ethanol evaporation.

Subsequently, the fruits in each group (*n* = 10) were divided into two microplates (each with 12 wells). Two milliliters of sterilized distilled water were added to each of the six wells in the front and back rows to maintain moisture and promote fungal growth. The contact point of the pedicel and each fruit was inoculated with 5 µL of an *A. flavus* IFM 47798 spore suspension (4.6 × 10^6^/mL). The microplates were wrapped with parafilm, with two pieces of autoclaved sponge placed between the cover and body to allow humid air circulation. They were incubated at 25 °C for 15 and 30 days (Figures S3 and S4). After incubation, the fruits were washed with ethanol:water (1:1 *v*/*v*), desiccated in an oven at 50 °C, ground, weighed, and analyzed by HPLC-FLD to determine AF contents.

To confirm the ability of *A. flavus* mycelia to penetrate the seeds of intact jujube fruits and accumulate AFs, GBG fruits (mid-mature stage) that showed high susceptibility to AF contamination in Section 2.1 were selected. The fruits (*n* = 12) were collected, sterilized, inoculated as described above, and incubated for 30 days. They were then divided into combined exocarp and mesocarp parts and seed parts, desiccated at 50 °C, ground, weighed, and analyzed by HPLC-FLD to determine the AF contents.

To investigate the fluctuation in AF levels in the seeds of intact fruits, GBG fruits (mid-mature stage) were divided into three subgroups (Figure S2): GBG-A (fruits with pedicels), GBG-B (fruits without pedicels), and GBG-C (fruits without pedicels that were artificially damaged at the pedicel contact area). The exocarp and mesocarp were removed from all fruits, and the kernels were sterilized and treated to evaporate ethanol as described above. The kernels of each subgroup (*n* = 10) were divided into two microplates (each with 12 wells), prepared and inoculated as with intact fruits, and incubated at 25 °C for 5 and 10 days (Figures S5 and S6). The kernels were washed with ethanol:water (1:1 *v*/*v*), divided into endocarp and seed parts, desiccated separately at 50 °C, ground, weighed, and analyzed by HPLC-FLD to determine the AF contents.

As Section 2.3 showed that protective barriers do not directly influence AF level fluctuations in kernel seeds, new experiments were performed to confirm this. The GBG kernels (without pedicels) (*n* = 10) were sterilized as described above and dipped in an *A. flavus* IFM 47798 spore suspension (4.6 × 10^6^/mL) for 15 min. The kernels were placed in microplates, prepared as previously described, and incubated for 30 days. After incubation, the kernels were sterilized, divided into endocarp and seed parts, desiccated separately at 50 °C, ground, weighed, and analyzed by HPLC-FLD to determine the AF contents.

#### 5.1.2. Jujube Seeds

Seeds of GBG fruits were collected (*n* = 22). Sucrose-free Czapek–Dox agar (CZA) was prepared as previously described (Elamin et al., 2018) [27]. A 100 µL spore suspension (4.6 × 10^6^/mL) of *A. flavus* IFM 47798 was spread on the surface of the medium in a Petri dish (90 mm × 15 mm). The seeds were washed with ethanol:water (1:1 *v*/*v*), placed in sterilized beakers, and stored in a biosafety cabinet overnight for desiccation. Ten seeds were spread on the surface of uninoculated agar and incubated for 5 days at 25 °C. The other 12 seeds were placed on *A. flavus*-inoculated agar and incubated under the same conditions. After incubation, the seeds were pooled, washed with ethanol:water (1:1 *v*/*v*), desiccated in an oven at 50 °C overnight, examined by SEM, and analyzed by HPLC-FLD to determine the AF contents.

### 5.2. AF Quantification Using HPLC

Each whole intact fruit from the GG, GBG, BG, and DBG groups (*n* = 40); the combined exocarp and mesocarp part and seed of each GBG intact fruit (*n* = 24); the endocarp and seed of each GBG kernel (*n* = 60); and the seeds from the GBG fruits used in the microscopic study (*n* = 10) were ground (Wonder Crusher WC-3; Osaka Chemical Co., Ltd., Osaka, Japan), weighed, and mixed with 4 mL of acetonitrile:water:methanol (6:4:1, *v*/*v*/*v*). The mixture was vortexed for 5 min at room temperature. After centrifugation (4770× *g*, 10 min, 4 °C), 0.4 mL of the supernatant was diluted to 10 mL with phosphate-buffered saline (PBS) containing 0.01% Tween 20 (Kanto Chemical Co., Inc., Tokyo, Japan). The mixture was filtered through a glass-fiber filter (GA-100; Advantec Toyo Kaisha, Ltd., Tokyo, Japan) and transferred to an immunoaffinity column (Aflaking, Horiba, Ltd., Kyoto, Japan). The column was washed twice with 3 mL of PBS and 3 mL of water, then eluted with 3 mL of acetonitrile. The eluate was dried under N_2_ gas, mixed with 0.1 mL of water:acetonitrile (9:1, *v*/*v*) and 0.1 mL of trifluoroacetic acid, and vortexed for 15 min. After adding 0.3 mL of water:acetonitrile (9:1, *v*/*v*), the reaction mixture was filtered through a 0.2 µm syringe filter (Minisart^®^ RC 4; Sartorius Stedim Lab. Ltd., Stonehouse, UK) and analyzed by HPLC-FLD (Capcell Pak C_18_ UG 120 column, 250 × 4.6 mm inner diameter; Osaka Soda Co., Ltd., Osaka, Japan) using the same method as in our previous study [22]. The AF mixture standard solution (FUJIFILM Wako Chemicals, Osaka, Japan) was used to prepare calibration solutions for HPLC-FLD determination and recovery experiments. The retention times, limits of detection, and limits of quantification for AFB_1_ and AFB_2_ were 7.15 and 12.60 min, 0.1 and 0.1 µg/kg, and 0.25 and 0.25 µg/kg, respectively. The linear range for AFB_1_ and AFB_2_ was 0.078 to 25 µg/kg, with a correlation coefficient (R^2^) greater than 0.9946. The samples that were outside the calibration range were diluted and reanalyzed to ensure accurate measurement.

### 5.3. SEM

SEM was performed using a TM3030 tabletop microscope (Hitachi, Ltd., Tokyo, Japan) under high vacuum. Images were captured in backscattered electron mode at 15 kV with a 5.00 mm working distance. SEM was used to observe the hilar region’s shape in the control and artificially contaminated seeds and to assess its permeability to fungal mycelia in artificially contaminated GBG jujube seeds.

## Figures and Tables

**Table 1:** AF concentrations of intact fruits exposed to *A. flavus* for 15 and 30 days.

Group	Rep.	15 Days of Incubation		Rep.	30 Days of Incubation	
		AFB_1_ (µg/kg)	AFB_2_ (µg/kg)		AFB_1_ (µg/kg)	AFB_2_ (µg/kg)
GG	1	<0.25 ^(b)^	ND ^(a)^	1	ND	ND
	2	1.0	ND	2	ND	ND
	3	ND	ND	3	ND	ND
	4	ND	ND	4	ND	ND
	5	<0.25	ND	5	<0.25	ND
GBG	1	14.6	<0.25	1	31.4	0.8
	2	2.3	ND	2	11.7	<0.25
	3	3499	87.7	3	<0.25	ND
	4	6.7	<0.25	4	<0.25	ND
	5	0.7	ND	5	0.3	ND
BG	1	<0.25	ND	1	ND	ND
	2	<0.25	ND	2	ND	ND
	3	<0.25	ND	3	0.5	ND
	4	<0.25	ND	4	52.0	1.0
	5	ND	ND	5	0.5	ND
DBG	1	ND	ND	1	ND	ND
	2	ND	ND	2	ND	ND
	3	ND	ND	3	ND	ND
	4	ND	ND	4	ND	ND
	5	ND	ND	5	ND	ND

^(a)^ below the LOD (0.1 μg/kg). ^(b)^ below the LOQ (0.25 μg/kg).

**Table 2:** AF concentrations of the two parts of jujube fruits when the intact fruits were exposed to *A. flavus* for 30 days and then divided.

Rep.	Part	AFB_1_ (µg/kg)	AFB_2_ (µg/kg)
1	exocarp + mesocarp	1819.0	42.1
	seed	1.8	ND ^(a)^
2	exocarp + mesocarp	1.1	ND
	seed	1.8	ND
3	exocarp + mesocarp	1.1	ND
	seed	ND	ND
4	exocarp + mesocarp	ND	ND
	seed	<0.25 ^(b)^	ND
5	exocarp + mesocarp	<0.25	ND
	seed	ND	ND
6	exocarp + mesocarp	0.66	ND
	seed	ND	ND
7	exocarp + mesocarp	3.9	ND
	seed	ND	ND
8	exocarp + mesocarp	<0.25	ND
	seed	ND	ND
9	exocarp + mesocarp	<0.25	ND
	seed	ND	ND
10	exocarp + mesocarp	0.4	ND
	seed	ND	ND
11	exocarp + mesocarp	<0.25	ND
	seed	0.3	ND
12	exocarp + mesocarp	1.4	ND
	seed	ND	ND

^(a)^ below the LOD (0.1 μg/kg). ^(b)^ below the LOQ (0.25 μg/kg).

**Table 3:** AF concentrations of endocarp and seed part of kernels after inoculation with *A. flavus* for 5 and 10 days.

Incubation Period	Rep.	Part	GBG-A		GBG-B		GBG-C	
			AFB_1_ (µg/kg)	AFB_2_ (µg/kg)	AFB_1_ (µg/kg)	AFB_2_ (µg/kg)	AFB_1_ (µg/kg)	AFB_2_ (µg/kg)
5 days	1	Endocarp	<0.25 ^(a)^	ND ^(b)^	2.3	0.3	1.7	ND
		Seed	ND	ND	14.1	ND	ND	ND
	2	Endocarp	1.3	<0.25	1.8	<0.25	1.8	ND
		Seed	ND	ND	ND	ND	ND	ND
	3	Endocarp	<0.25	ND	1.1	ND	1.3	ND
		Seed	ND	ND	ND	ND	1.9	ND
	4	Endocarp	0.4	ND	0.9	ND	1.7	ND
		Seed	ND	ND	ND	ND	ND	ND
	5	Endocarp	4.8	0.8	1.1	ND	1.9	ND
		Seed	ND	ND	ND	ND	ND	ND
10 days	1	Endocarp	<0.25	ND	2.3	ND	2.2	<0.25
		Seed	ND	ND	ND	ND	ND	ND
	2	Endocarp	1.4	ND	2.9	ND	1.6	ND
		Seed	ND	ND	ND	ND	990	23.9
	3	Endocarp	0.7	ND	2.1	ND	2.7	ND
		Seed	ND	ND	ND	ND	ND	ND
	4	Endocarp	1.7	ND	5.3	ND	2.1	ND
		Seed	4605	46	ND	ND	ND	ND
	5	Endocarp	1.8	ND	2.4	ND	2.3	<0.25
		Seed	14.2	ND	ND	ND	ND	ND

^(a)^ below the LOQ (0.25 μg/kg). ^(b)^ below the LOD (0.1 μg/kg).

**Table 4:** AF concentrations of the kernels’ seeds when the jujube kernels were dipped in the spore suspension of *A. flavus* and incubated for 30 days.

Rep.	AFB_1_ (µg/kg)	AFB_2_ (µg/kg)
1	1.0	ND ^(a)^
2	0.7	ND
3	ND	ND
4	<0.25	ND
5	ND	ND
6	ND	ND
7	ND	ND
8	ND	ND
9	21,572	535
10	0.7	ND

^(a)^ below the LOD (0.1 μg/kg).

**Table 5:** AF concentrations of the seeds inoculated with *A. flavus*.

Seed (Figure No.)	HF Status	AFB_1_ (µg/kg)	AFB_2_ (µg/kg)
2A	nearly closed	3.4	ND ^(a)^
2B		4.9	ND
2C		7.8	ND
2D		2.8	ND
2E		3.1	ND
2F		8.0	ND
2G	Semi-closed	24.7	ND
2H		111.2	0.8
2I		8.7	ND
2J		9.8	ND
2K		17.9	ND
2L	opened	118,456	1754

^(a)^ below the LOD (0.1 μg/kg).

## Data Availability

The original contributions presented in this study are included in this article and Supplementary Materials. Further inquiries can be directed to the corresponding author.

## Supplementary Materials

The following supporting information can be downloaded at: https://www.mdpi.com/article/10.3390/toxins17030113/s1, Figure S1. The four groups of jujube fruits. Figure S2. Artificially prepared samples of the three subgroups. Figure S3. The jujube fruits were inoculated with the spores of *A. flavus* and incubated for 15 days at 25 °C. Figure S4. The jujube fruits were inoculated with the spores of *A. flavus* and incubated for 30 days at 25 °C. Figure S5. The sample of subgroups GBGA–C before and after 5 days of incubation. Figure S6. The sample of subgroups GBGA–C before and after 10 days of incubation.
