# Supplementary material for: Susceptibility and Mechanism of Aflatoxin Contamination of Ziziphus jujuba var. spinosa
Source: Toxins (Basel). 2025 Feb 27;17(3):113. doi: 10.3390/toxins17030113 (PMC11946733; doi:10.3390/toxins17030113)
Supplement: Supplementary file 1 [file toxins-17-00113-s001.zip › toxins-3467950-supplementary.pdf]

Supplementary Materials

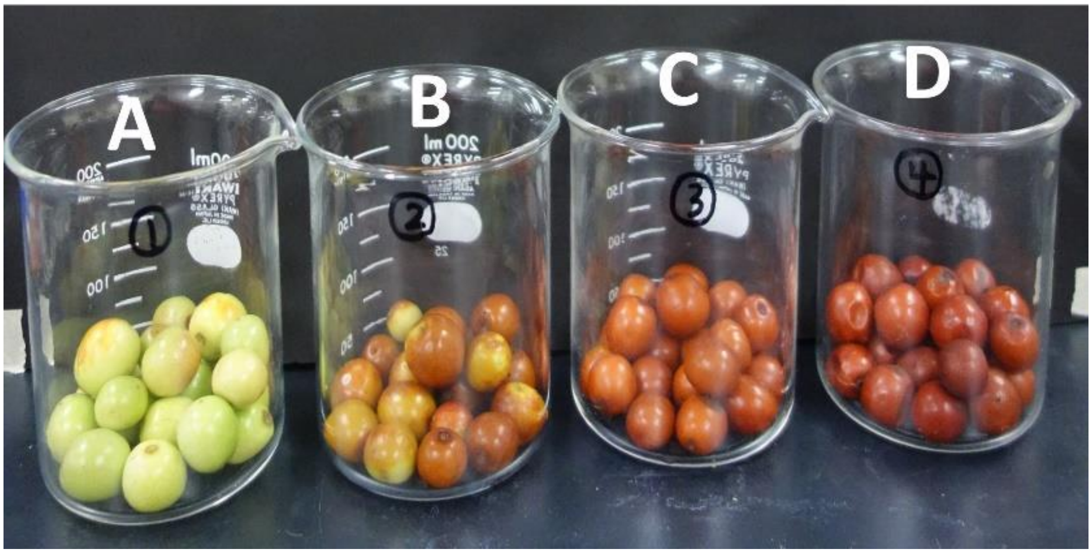

Figure S1. The four groups of jujube fruits. A. GG fruits. B. GBG fruits. C. BG fruits. D. DBG fruits.

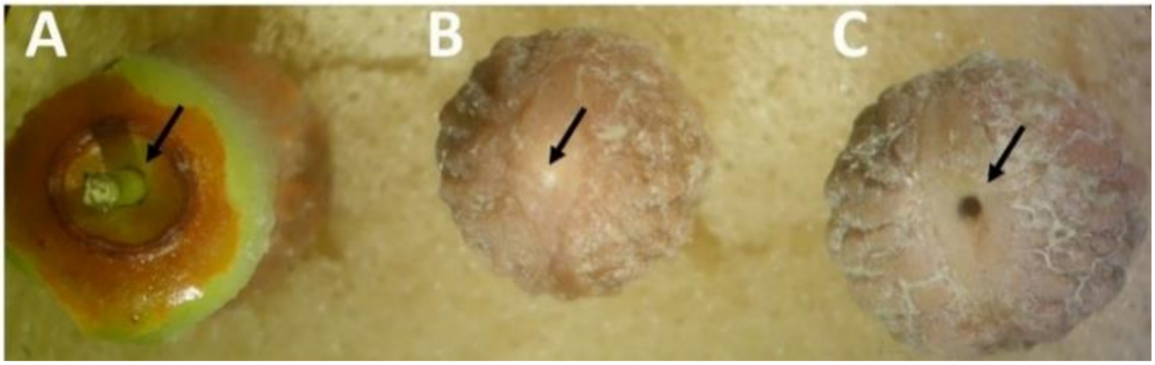

Figure S2. Artificially prepared samples of the three subgroups. A. GBG-A sample (with pedicel). B. GBG-B sample (without pedicel). C. GBG-C sample (without pedicel and artificially damaged). Black arrows indicate the point of contact between the pedicel and kernel.

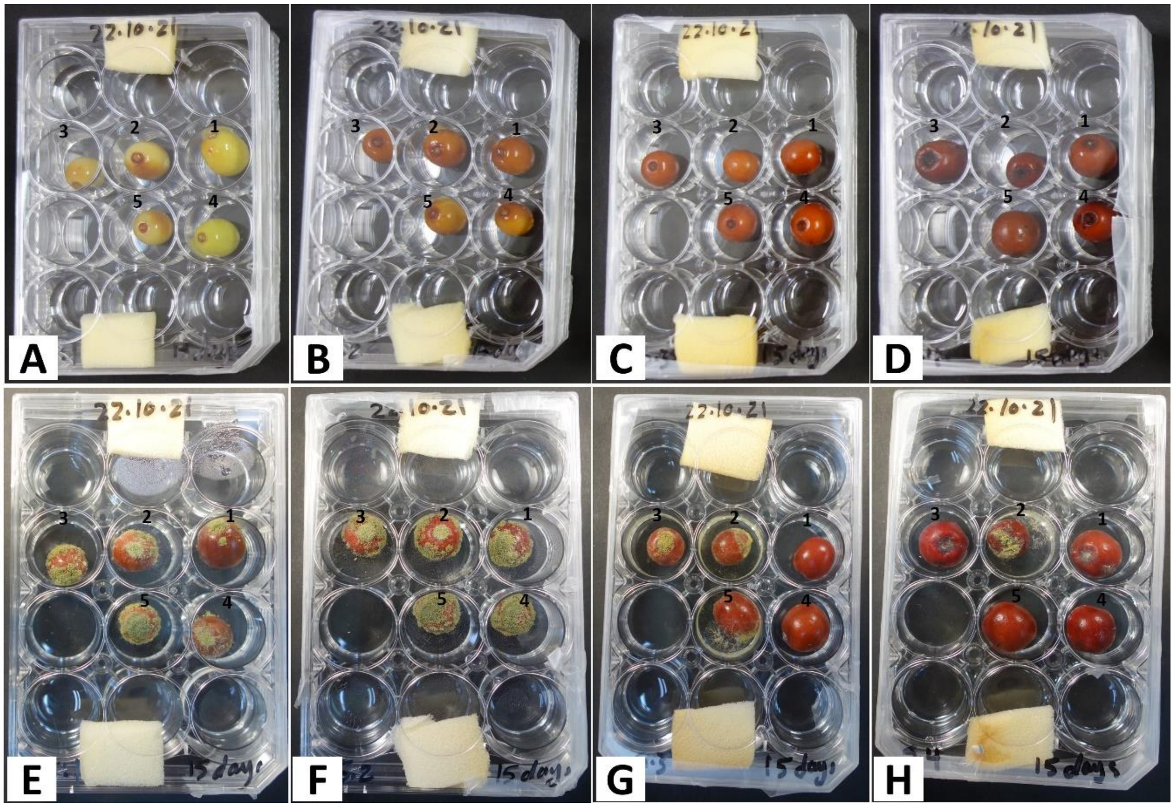

Figure S3. The jujube fruits were inoculated with the spores of *A. flavus* and incubated for 15 days at 25 ˚C. (A) and (E) fruits of the GG before and after incubation. (B) and (F) fruits of the GBG before and after incubation. (C) and (G) fruits of the BG before and after incubation. (D) and (H) fruits of the DBG before and after incubation.

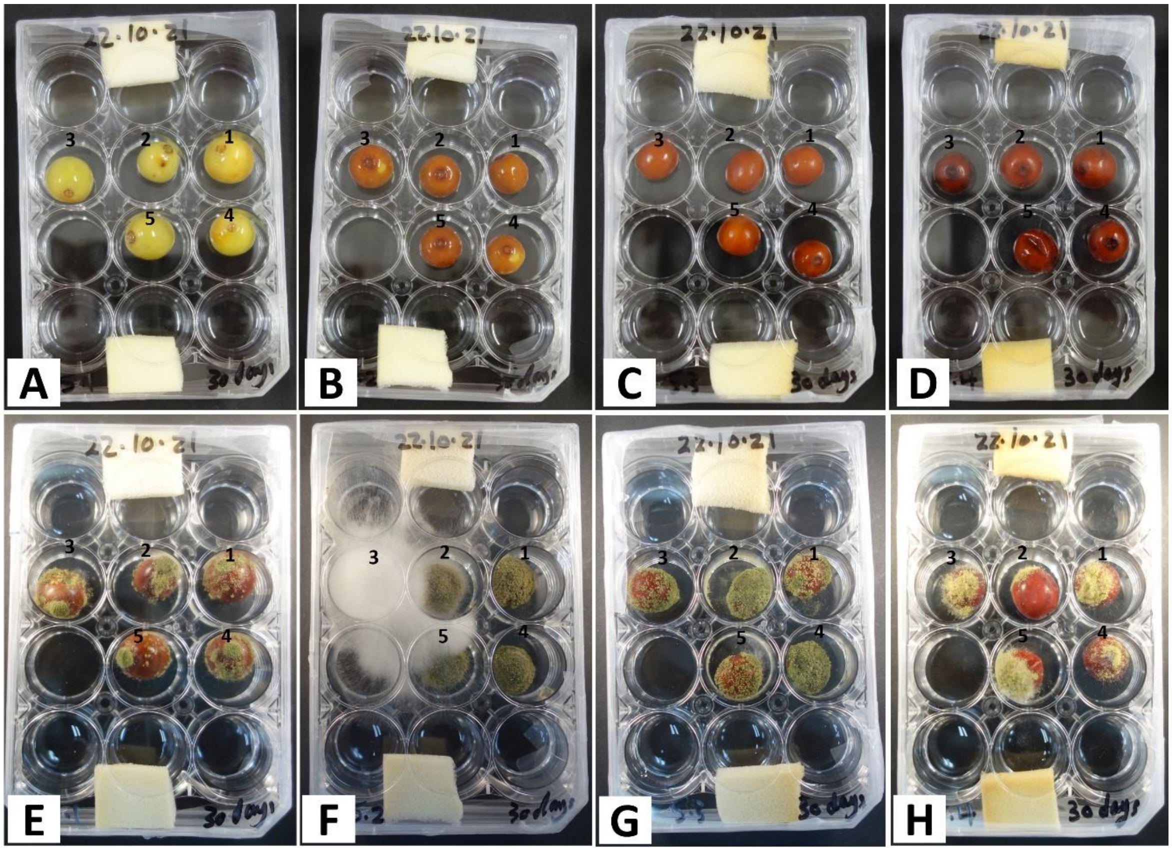

Figure S4. The jujube fruits were inoculated with the spores of *A. flavus* and incubated for 30 days at 25 ˚C. (A) and (E) fruits of the GG before and after incubation. (B) and (F) fruits of the GBG before and after incubation. (C) and (G) fruits of the BG before and after incubation. (D) and (H) fruits of the DBG before and after incubation.

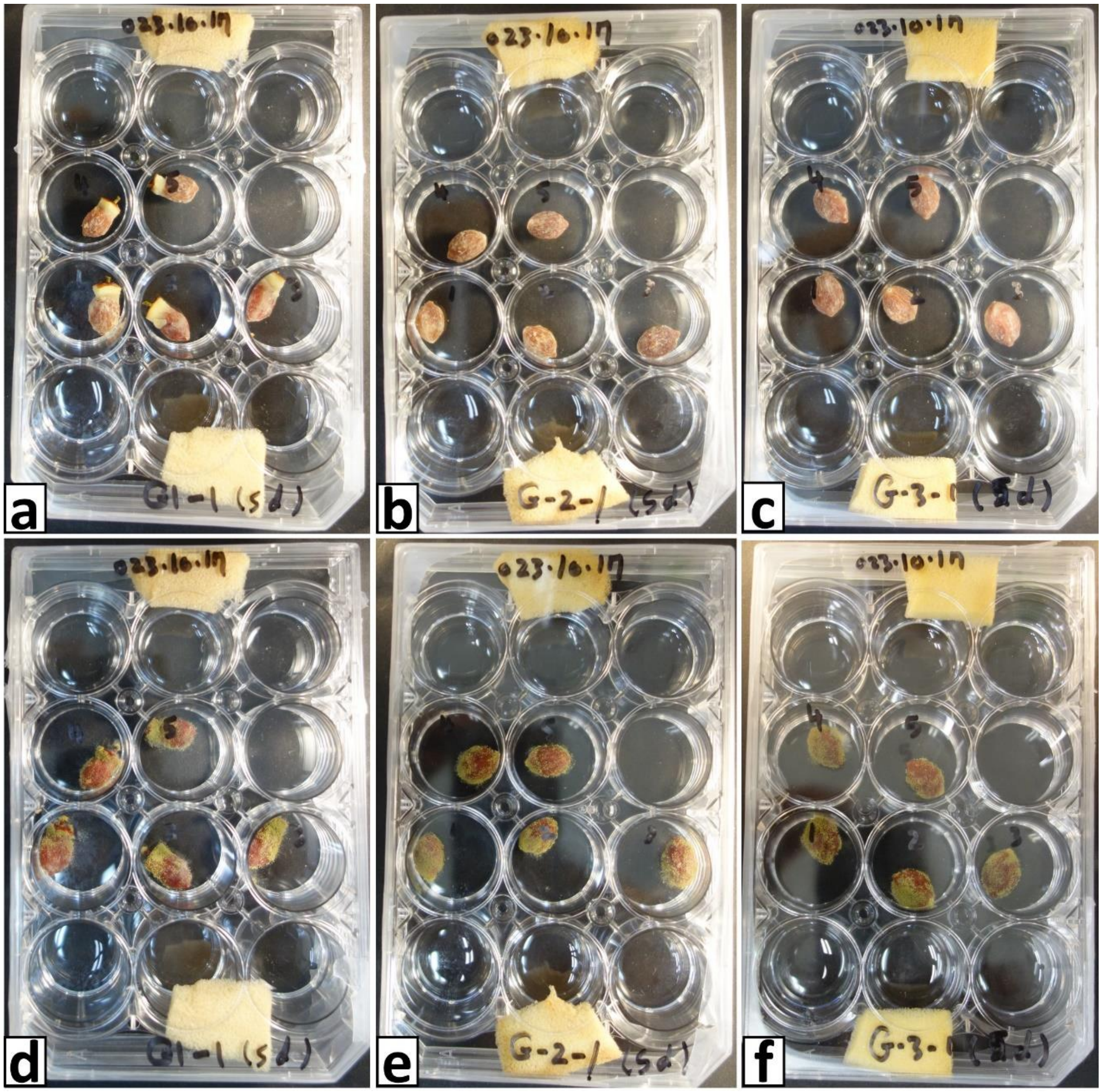

Figure S5. The sample of subgroups GBGA–C before and after 5 days of incubation. (a-c) samples of the GBGA–C before incubation, respectively. (d-f) samples of the GBGA–C after incubation, respectively.

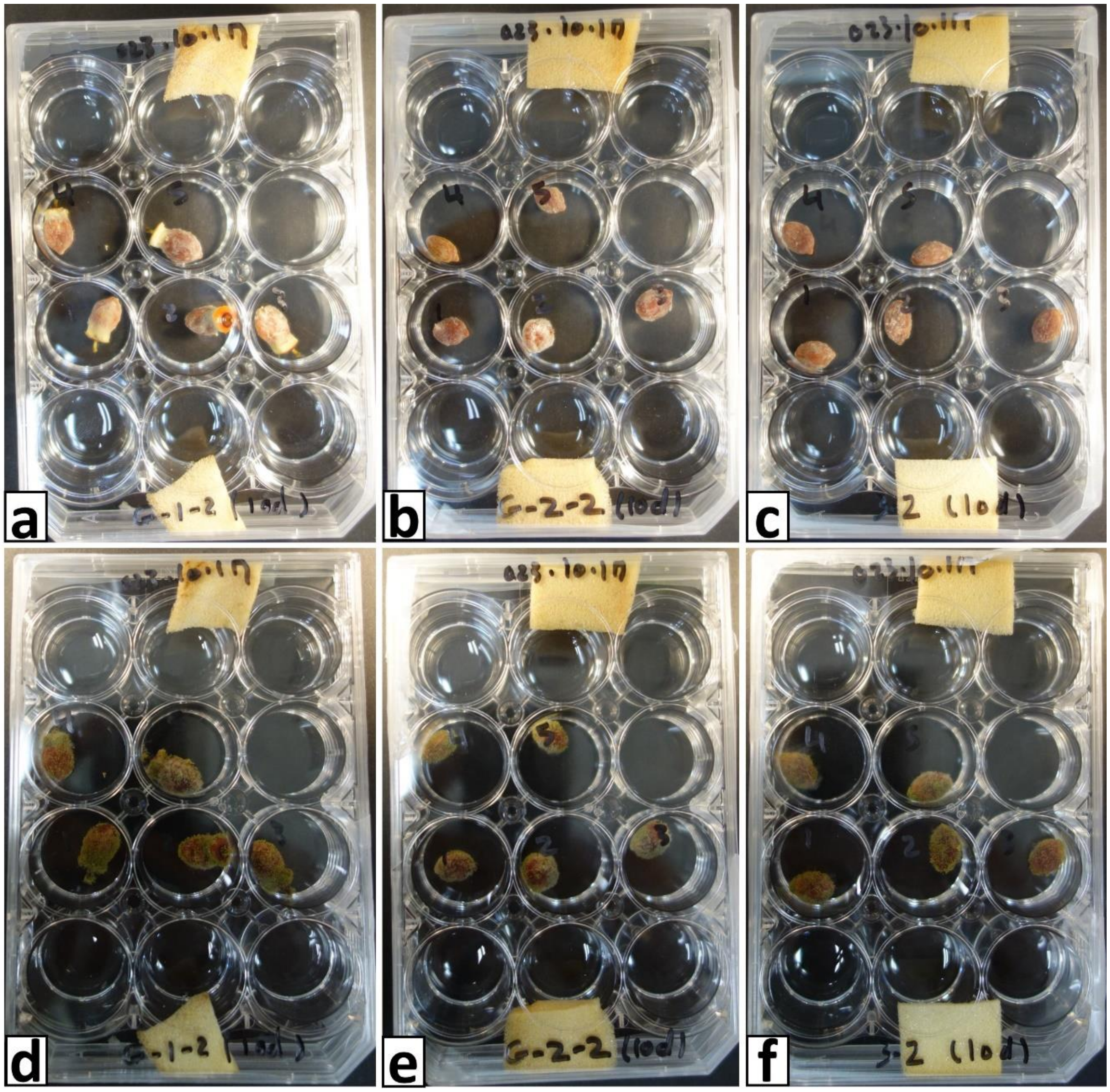

Figure S6. The sample of subgroups GBGA–C before and after 10 days of incubation. (a-c) samples of the GBGA–C before incubation, respectively. (d-f) samples of the GBGA–C after incubation, respectively.
